# Prevalence and Scanning Electron Microscopic Identification of Anoplocephalid Cestodes among Small Ruminants in Senegal

**DOI:** 10.1155/2016/3937292

**Published:** 2016-08-11

**Authors:** Mallé Ndom, Gora Diop, Yann Quilichini, Tetsuya Yanagida, Cheikh Tidiane Ba, Bernard Marchand

**Affiliations:** ^1^Laboratoire de Biologie Évolutive, d'Écologie et de Gestion des Écosystèmes, Faculté des Sciences et Techniques, Université Cheikh Anta Diop de Dakar, BP 5055, Dakar, Senegal; ^2^CNRS, Université de Corse, UMR SPE 6134, Service d'Étude et de Recherche en Microscopie Électronique, Campus Grimaldi, BP 52, Corte, 20250 Corse, France; ^3^Laboratory of Veterinary Parasitology, Joint Faculty of Veterinary Medicine, Yamaguchi University, Yoshida 1677-1, Yamaguchi 753-8515, Japan

## Abstract

This study was undertaken to determine the prevalence of anoplocephalid cestodes in sheep and goats in Senegal. Intestines of 462 sheep and 48 goats were examined; 47.4% of sheep and 6.2% of goats were infected. The species identified and their prevalence were, among sheep,* Avitellina centripunctata* 38.7%,* Moniezia expansa* 15.4%,* Stilesia globipunctata* 16.7%, and* Thysaniezia ovilla* 0.4%. Among goats, they were* M. expansa* 6.2% and* T. ovilla* 2.1%. The prevalence of all species was not statistically different between dry and rainy seasons. The infections were single or multiple. Indeed, 56.2% of sheep were infected by a single species, 37.4% by two species, and 6.4% by three species. For goats, 66.7% were infected by* M. expansa* and 33.3% by both* M. expansa* and* T. ovilla*. Scanning electron microscopic (SEM) observations of tapeworms show the general diagnosis characters of these species.

## 1. Introduction

In Senegal, livestock play both social and economic roles. It remains as the only source of livelihood for 30% of rural households. It is indeed a great potential for wealth creation in the sense that it represents 4.2% of the national Gross Domestic Product and 28.8% of the primary sector (GDP) [1].

Small ruminants represent 69.3% of livestock. Nevertheless the enormous potential productivity of small ruminants is still weak, due to several constraints, including malnutrition, management, and diseases [1]. In fact, diseases caused by parasites are also factors that contribute to reduction of the productivity of these animals around the world [2]. This is also a reality in sub-Saharan regions [3]. Gastrointestinal helminthes, with their toxic, mechanical, and absorptive actions, constitute a major constraint to health and productivity of domestic animals, especially in developing countries [4–7].

As far as we know, in Africa, five species of anoplocephalid cestodes have been reported as major intestinal infections in small ruminants [8, 9]. These are* Avitellina centripunctata* Rivolta, 1874,* Moniezia expansa* Rudolphi, 1810,* Moniezia benedeni* Moniez, 1879,* Stilesia globipunctata* Rivolta, 1874, and* Thysaniezia ovilla* Rivolta, 1878, with a prevalence ranging from 6 to 92% [10–19]. In Senegal, these species have been confirmed, but their monthly and seasonal dynamics in ruminants have not yet been elucidated [20].

The aims of present study were to determine the prevalence of the anoplocephalid cestodes infecting sheep and goats in Senegal throughout the year and after performing scanning electron microscopic (SEM) observations of specimens to put into evidence the characteristic of these species.

## 2. Materials and Methods

### 2.1. Ruminant Hosts, Cestode Collection

Small intestines of a total of 462 sheep (*Ovis aries*) and 48 goats (*Capra hircus*) were collected from the main slaughterhouse of Dakar, Senegal, from January to December, 2013. Sampling periods were characterized by a rainy season (500–900 mm/year) and a dry season extending from June to October and November to May, respectively. Ruminant hosts were mostly between 2 and 5 years old, and they came from different localities in Senegal. Small intestines were examined to recover adult cestodes.

### 2.2. Staining Identification and SEM Study

The cestodes were collected carefully from the intestines of small ruminants (sheep/goats) and identified after staining, using keys [21–24]. A carmine stain was on mature proglottids. Proglottids were fixed and washed in 70% ethanol. Staining was with iron hydrochloric carmine, destained in acid ethanol (100 mL 70% ethanol + 2 mL concentrated HCl), dehydrated in a graded ethanol series, cleared with eugenol (clove oil), and mounted in Canada balsam. Stained specimens were examined and photographed under a Leitz photo research microscope. To confirm detailed surface morphology, SEM observations were performed. For SEM, worms were isolated carefully from intestines and placed into a small amount of saline buffer. For living tapeworms, scolex, mature, and gravid proglottids of many specimens of each species were fixed overnight in cold 2.5% glutaraldehyde in a 0.1 M sodium cacodylate buffer at pH 7.4. Then, they were dehydrated in a graded ethanol series and dried using CO_2_ in an Emitech K850 critical point dryer. After being mounted on metal stubs, specimens were coated with gold/palladium in a Quorum Technologies SC7640 sputter coater and examined with a Hitachi S-3400N scanning electron microscope at acceleration voltages between 3 and 20 kV in the “Service d'Etude et de Recherche en Microscopie Electronique de l'Université de Corse.”

### 2.3. Statistical Analysis

The prevalence was calculated by dividing the number of animals harboring parasites by total number of examined animals. Mean intensity was assessed by dividing the total number of parasites by the number of infected hosts. Statistical analyses were performed using R software [25]. Percentages (%) measure prevalence and chi-square (*χ* ^2^) association between prevalence of the parasite and species. In all the analyses, confidence levels were 95% and significance at *p* < 0.05.

## 3. Results

### 3.1. Prevalence and Dynamic of Anoplocephalid Infections

Among the examined animals, 219 sheep and three goats were infected, corresponding to a prevalence of 47.4% and 6.2%, respectively. Among sheep, the total number of parasites was 1304 and mean intensity was 5.9 (range 1–40). Among goats total number of parasites was 7, with a mean intensity of 2.3 (range 1–5). Four species of anoplocephalid cestodes were recorded:* Avitellina centripunctata, Moniezia expansa, Stilesia globipunctata*, and* Thysaniezia ovilla*. In this study, 38.7% of sheep were infected by* A. centripunctata*, 15.4% by* M. expansa*, 16.7% by* S. globipunctata*, and 0.4% by* T. ovilla*. For goats, 6.2% were infected by* M. expansa* and 2.1% by* T. ovilla*.

**Figure 1:**
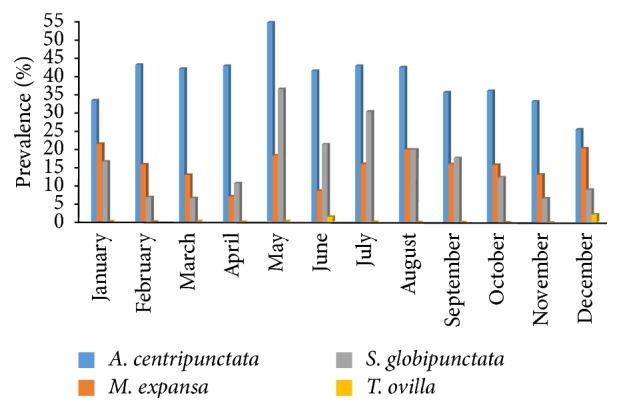
Monthly variation of prevalence of anoplocephalids in sheep.

**Figure 2:**
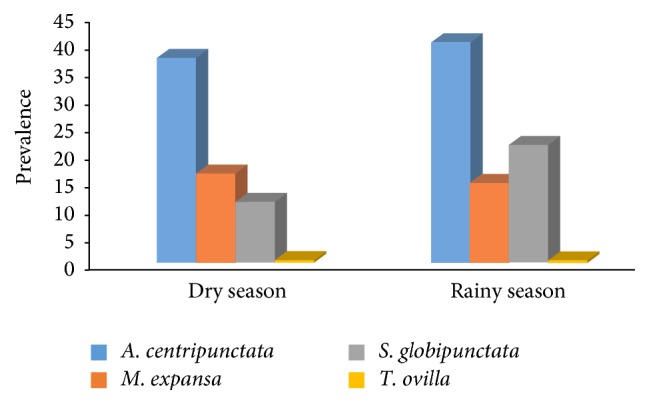
Seasonal variation of prevalence of anoplocephalids in sheep. Dry season extends from November to May, while rainy season extends from June to October.

Monthly and seasonal dynamics of prevalence were shown in Figures 1 and 2, respectively. The highest infection rates were recorded during May for* A. centripunctata* (54.5%), January for* M. expansa* (21.4%), and May for* S. globipunctata* (36.4%). The lowest value was noted in December for* A. centripunctata* (25.4%), April for* M. expansa* (7%), and March for* S. globipunctata* (6.5%). For each species, the differences on prevalence were not statistically significant between dry season and rainy season: *p* value = 0.94 for* A. centripunctata*, 0.64 for* M. expansa*, 0.28 for* S. globipunctata*, and 0.93 for* T. ovilla*. The mean intensity of infection for the tapeworms was 5.1, 4.2, 3.4, and 1.5 for* S. globipunctata, M. expansa, A. centripunctata*, and* T. ovilla*, respectively, and was not statically different between dry and rainy season (Table 1).

Single and associated species infections were evaluated among both sheep and goats. Among sheep, the infections due to one, two, or three species were 56.2%, 37.4%, and 6.4%, respectively. For the infections caused by one species,* A. centripunctata* was the most prevalent with 39.3% followed by* M. expansa* (13.2%) and* S. globipunctata* (3.6%). For the infections caused by two species,* A. centripunctata* and* S. globipunctata* were more prevalent (24.2%) than* A. centripunctata* and* M. expansa* (11.4%),* M. expansa* and* S. globipunctata* (1.4%), and* A. centripunctata *and* T. ovilla* (0.5%). As for the infections due to three species,* A. centripunctata, M. expansa*, and* S. globipunctata* were more prevalent with 5.9% than* A. centripunctata, M. expansa*, and* T. ovilla* (0.5%). Among goats, two of infected animals harbored a single species* M. expansa*, and the remaining harbored* M. expansa* and* T. ovilla*.

### 3.2. Scanning Electron Microscopy

**Figure 3:**
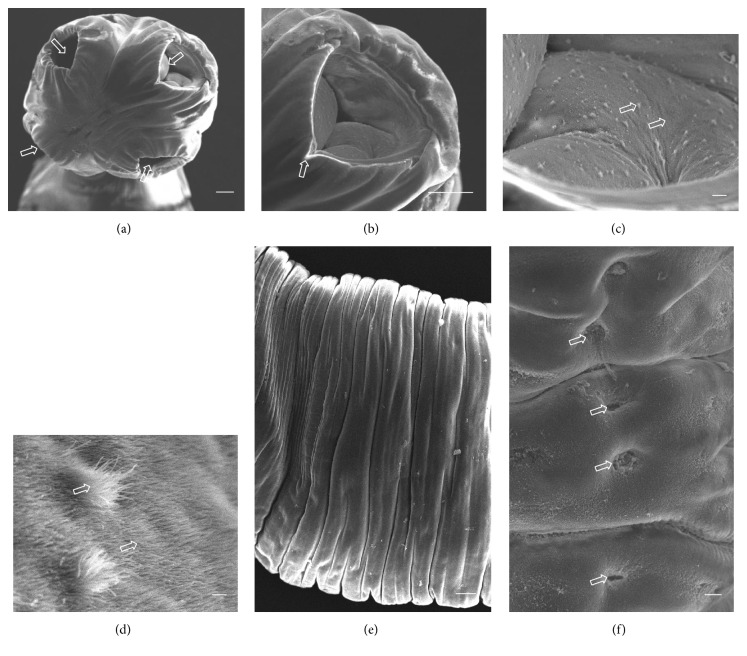
(a)–(f) Scanning electron micrographs of* Avitellina centripunctata*. (a) General anterior view of scolex, arrows show invaginated suckers. (b) Invaginated sucker, arrow showing triangular opening. (c) Peripheral sucker, arrows show acicular and capilliform filitriches (Scale bar, 10 *μ*m). (d) Acicular and capilliform filitriches covering sucker (scale bar, 1 *μ*m). (e) Portion of strobila showing acraspedote proglottids. (f) Lateral portion of strobila, arrows show genital pores (Scale bar, 10 *μ*m). Scale bar = 100 *μ*m, unless stated otherwise.

**Figure 4 fig4:**
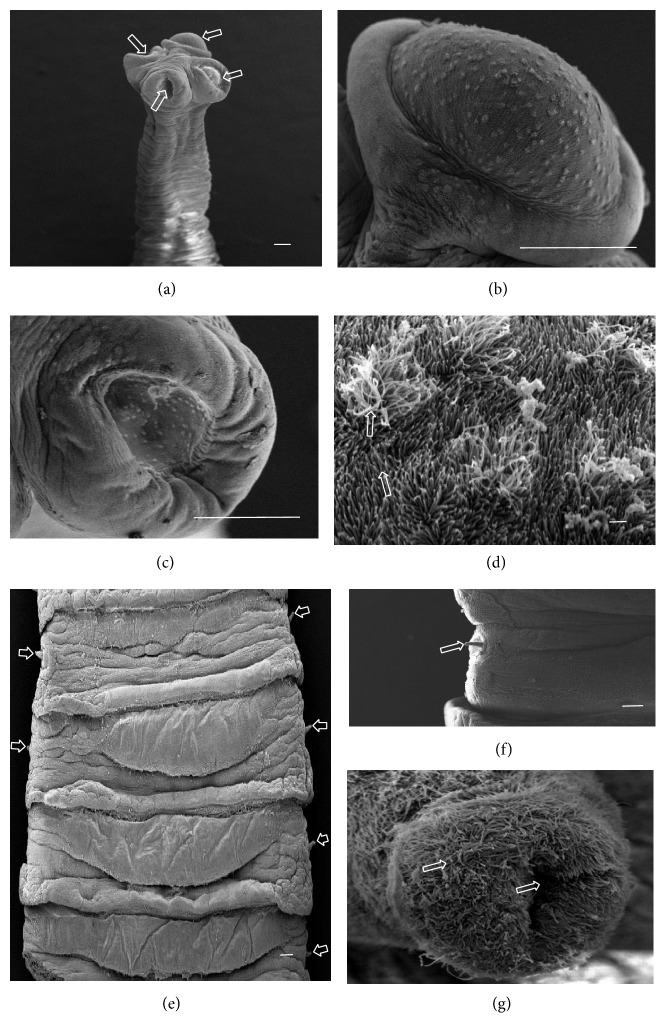
(a)–(g) Scanning electron micrographs of* Moniezia expansa*. (a) General anterior view of scolex, arrows show suckers. (b) Sucker evaginated. (c) Sucker invaginated. (d) Acicular and capilliform filitriches covering suckers (scale bar, 1 *μ*m). (e) Portion of strobila showing craspedote proglottids and bilateral genital pores. (f) Portion of proglottid, arrow shows cirrus. (g) Cirrus fully covered by microtriches and showing cavity in center (scale bar, 1 *μ*m). Scale bar = 100 *μ*m, unless stated otherwise.

**Figure 5 fig5:**
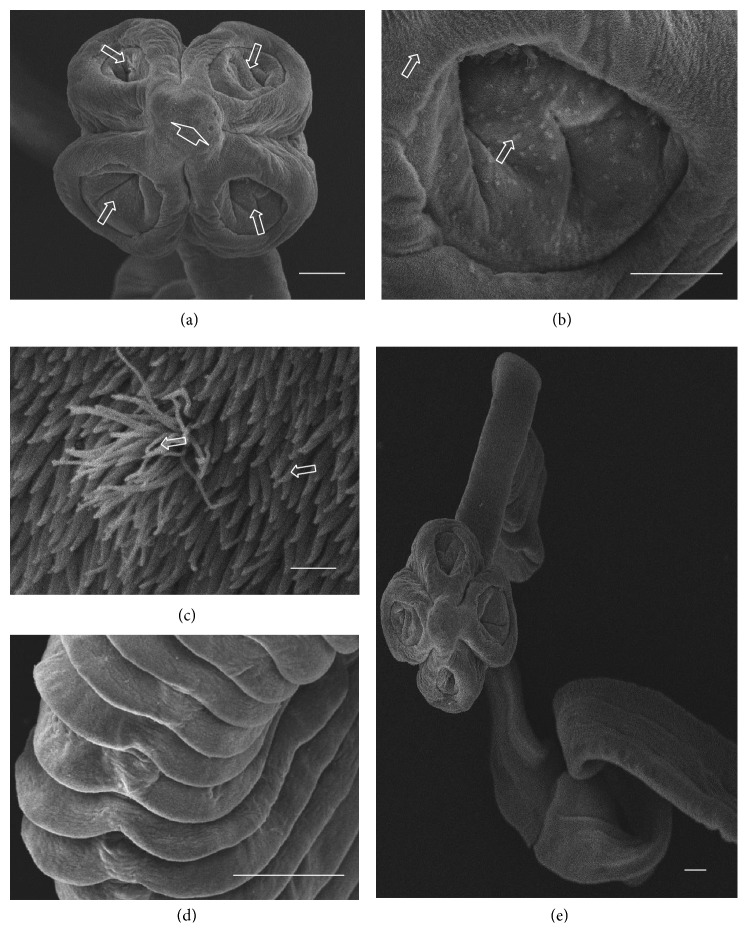
(a)–(e) Scanning electron micrographs of* Stilesia globipunctata*. (a) General anterior view of scolex, arrows show invaginated suckers and arrowhead shows apical part. (b) Invaginated sucker, arrows show acicular and capilliform filitriches (scale bar, 50 *μ*m). (c) Acicular and capilliform filitriches covering sucker (scale bar, 1 *μ*m). (d) Portion of* S. globipunctata* showing spiral strobila. (e) Portion of strobila showing craspedote proglottids. Scale bar = 100 *μ*m, unless stated otherwise.

**Figure 6:**
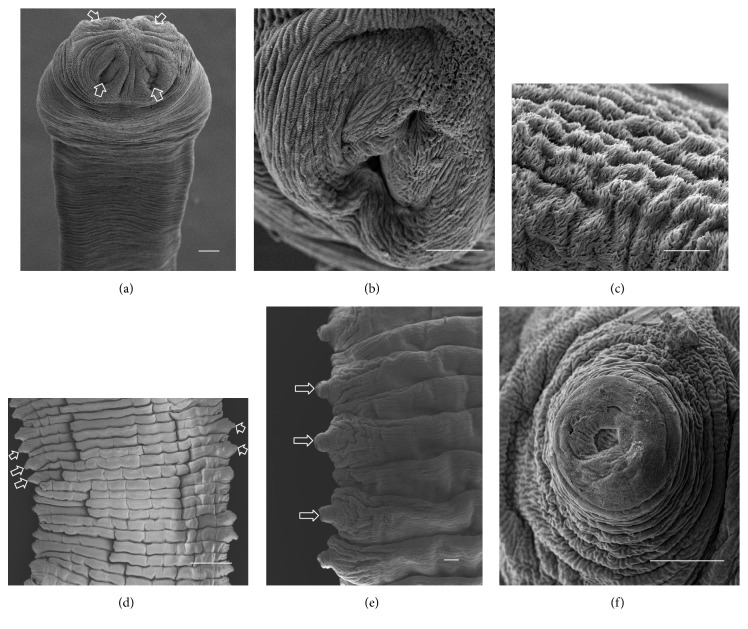
(a)–(f) Scanning electron micrographs of* Thysaniezia ovilla*. (a) General anterior view of scolex, arrows show invaginated suckers. (b) Invaginated sucker (scale bar, 50 *μ*m). (c) Peripheral sucker showing capilliform filitriches (scale bar, 10 *μ*m). (d) Portion of strobila showing genital pores irregularly altering (Scale bar, 1 *μ*m). (e) Portion of proglottids, arrows show cirrus. (f) Cirrus apical view (scale bar, 50 *μ*m). Scale bar = 100 *μ*m, unless stated otherwise.

A total of ten* A. centripunctata*, six* S. globipunctata*, six* M. expansa*, and six* T. ovilla* were examined. All* Avitellina* and* Stilesia* and four* Moniezia* and* Thysaniezia* specimens were collected from sheep; two* Moniezia* and* Thysaniezia* specimens were collected from goats. The results of scanning on specimen of each species were presented in Figures 3 [Fig fig4] [Fig fig5]–6.

#### 3.2.1. *A. centripunctata*

The scolex is unarmed, spherical in shape, 1000 to 1600 *μ*m in diameter, and provided with four suckers located at its peripheral margin (Figure 3(a)). Each sucker shows a triangular opening from which the point of the triangle is directed towards the center of the scolex (Figure 3(b)). The tegument of the scolex, suckers, and strobila of species are covered with acicular and capilliform filitriches (Figures 3(c) and 3(d)). The strobila has a threadlike appearance and is acraspedote, showing unilateral genital pores irregularly alternating. Proglottids are wider than longer and weakly distinguished even in mature proglottids (Figures 3(e) and 3(f)).

#### 3.2.2. *M. expansa*

The scolex is unarmed, globular in shape, 400 to 600 *μ*m in diameter, and provided with four suckers located at its peripheral margin (Figure 4(a)). Each sucker shows a circular or subcircular opening (Figures 4(b) and 4(c)). The strobila has a striped appearance and is craspedote. The proglottids are wider than longer. The genital pores are paired in each proglottid and disposed laterally (Figures 4(e) and 4(f)). The tegument of the scolex, suckers, and strobila are covered with acicular and capilliform filitriches (Figure 4(d)). The cirrus is entirely covered by capilliform filitriches (Figures 4(f) and 4(g)).

#### 3.2.3. *S. globipunctata*

The scolex is unarmed and is club-shaped, 300 to 600 *μ*m in diameter, and provided with four well-developed suckers (Figure 5(a)). Each sucker shows a circular opening (Figure 5(b)). At the center of the scolex, a rounded structure appears (Figure 5(a)). The unilateral genital pores are irregularly alternating. The tegument of scolex, suckers, and strobila is covered with acicular and capilliform filitriches (Figure 5(c)). Strobila is craspedote, threadlike, and very spiral (Figures 5(d) and 5(e)).

#### 3.2.4. *T. ovilla*

The scolex is unarmed and round, 300 to 450 *μ*m in diameter, and provided with four well-developed suckers (Figure 6(a)). Each sucker shows a circular opening (Figure 6(b)). The strobila has a striped appearance and is craspedote. The tegument of the scolex, suckers, and strobila is covered with capilliform filitriches (Figure 6(c)). The proglottids are wider than longer with unilateral genital pores irregularly alternating (Figure 6(d)). From the genital pores, a rounded cirrus protrudes, which exhibits a cavity in the center (Figure 6(f)).

## 4. Discussion

Infections by anoplocephalid cestodes are common among sheep and goats [17, 19, 26–29]. In Senegal, five species of Anoplocephalidae,* A. centripunctata, M. expansa, M. benedeni, S. globipunctata*, and* T. ovilla*, have already been described among ruminants [11, 14, 26, 30]. In our study, all except* M. benedeni* were found among sheep, but among goats only* M. expansa* and* T. ovilla* have been observed. The overall prevalence of anoplocephalids was significantly higher among sheep than among goats, as previously reported in Senegal by Ba et al. [14] and Ndao et al. [31]. Other studies in west African countries such as Gambia, Burkina Faso, and Côte d'Ivoire showed similar prevalence to those obtained in our study and confirmed that sheep were more frequently infected than goats [15, 17, 27]. The behavior of goats in pasture seems to limit their risk of infections and could explain also their low infestation intensity. Goats consume longer pasture than sheep, making intermediate oribatid mite hosts less available; another fact is that goats consume more ligneous food, rich in tannins, which are known natural anthelmintics and probably limit the infection [17].

The epidemiology of anoplocephalid infection depends on geographical localities. Our study and others showed that infection in sheep is generally higher in western African areas compared to others: in North Africa such as Egypt (11%) and in some European areas such as Turkey (4.4%) [19, 32]. The prevalence among goats was slightly lower in Turkey (6.3%) [33] compared to previous reports in Senegal [14, 31]. These differences can be attributed to climatic factors that are often highly variable from one area to another, such as the duration of the rainy season, which may affect the dynamics of intermediate hosts (oribatid mites). Also the soil kind, as oribatids, prefers acid soils and the type of farming in the different localities may therefore affect the prevalence of anoplocephalids [11].

In the previous studies of prevalence of anoplocephalids among small ruminants,* M. expansa* was the most prevalent [26, 31]. Nevertheless in our work,* A. centripunctata* was twice as prevalent as* M. expansa*. These facts can be probably explained by the different age-group of ruminants examined in the studies, because it is known that* A. centripunctata* is the more common among adult ruminants and* M*.* expansa* is the highest among one-year-old ruminants [13, 34]. Another fact is that* A. centripunctata* better resists the immune system of hosts than* M*.* expansa*. Sharkhuu [35] reported in Mongolia that* Moniezia* is common in goats and* Avitellina* and* Thysaniezia* are common in sheep.

In present study, the prevalence of* M. expansa* among sheep and goats are similar to those reported in Western Ethiopia [32], but lower than in Senegal [30, 31], Côte d'Ivoire [17], Eastern Ethiopia [36], and Guinea [29]. For* A. centripunctata*, the prevalence is lower than those reported in Senegal [18], similar to Côte d'Ivoire [17], and higher than in Senegal [14] and in Côte d'Ivoire [16]. In our study,* A. centripunctata* was not found among goats as previously reported in Côte d'Ivoire [16, 17] and Mongolia [35] but in Senegal Ba et al. [14] have reported 8% in goats. For* S. globipunctata*, the prevalence in sheep in our study was similar to that in Côte d'Ivoire [17], but higher than in Senegal [18].* S. globipunctata* was also not found among goats in this study. The same result was reported previously in Côte d'Ivoire [16], Guinea [29], and Western Ethiopia [32]. For* T. ovilla*, the prevalence was low, similar to the data from Sharkhuu [35], but different from reports by Nadège [18] who found* T. ovilla* from sheep in Senegal, with a prevalence of 84%.

Our study shows generally a decrease of anoplocephalid prevalence among small ruminants in Senegal. This reduction may be due to different control practices. Since 1996 with the National Plan for the Development of Animal Husbandry (NPDAH) and its strategy of contributing to improving health and zootechnical conditions of livestock, the Senegalese state has opted for a development of private veterinary advice to farmers. A strategic deworming plan was generally proposed during the rainy season because of the heavy infestation of ruminants and at the end of the dry season for their weakness [18].

Other investigations have shown that climate is an important factor in determining levels of infection by anoplocephalids. Generally anoplocephalids are more prevalent in the rainy season than the dry season [15, 26]. The probable reasons include higher humidity and temperature for the development of intermediate hosts. Oribatids are susceptible to desiccation and this fact may be a reason for little infection during the warmer months [34]. However, our study revealed two peaks of prevalence both in the dry and in the rainy seasons unlike the previous reports. Except for* T. ovilla*, anoplocephalids were present in small ruminants all the year. The four anoplocephalids recorded did not show significant differences between rainy and dry seasons. This lack of difference between seasons can be explained by deworming control methods applied in both rainy and dry seasons. Also the mean intensity was generally low, due probably to the large size of these tapeworms exceeding generally one meter [21] and/or control methods by veterinaries services and breeders. No significant difference between dry and rainy season can be attributed to the longevity of the tapeworms, which exceed a year [34].

Multiple infections of anoplocephalids were reported previously in Senegal but were not fully assessed [26]. In the present study, the infection with* A. centripunctata* and* S. globipunctata* was the most prevalent (24.2%), followed by* A. centripunctata* and* M. expansa* (11.4%). The multiple infestations play an important role in growth retardation and weight loss of small ruminants [15]. More, it was reported that coinfections can be lethal among small ruminants [33, 36–39].

The anoplocephalid species have all been described using light microscopy. Only a few scanning studies have been made on* A. centripunctata, T. ovilla*, and* M. expansa*, and these previous studies focused only on the scolex [19, 40]. Thus anoplocephalids have an unarmed scolex with four well-developed suckers. Suckers have circular, oval, or linear openings, depending on the level of contraction. The proglottids are wider than longer; they are craspedote for* M. expansa, S. globipunctata*, and* T. ovilla* and acraspedote for* A. centripunctata*.* M. expansa* and* T. ovilla* are large worms compared to* A. centripunctata* and* S. globipunctata*. They differ in the presence of paired genitalia in* M. expansa* and single and irregularly alternating genital pores in* T. ovilla*.* S. globipunctata* and* A. centripunctata* have threadlike strobila.* S. globipunctata* is short and the strobila is spiral. The body of cestodes is fully covered by microtriches, presenting different forms and size among specimens. Then standardized terminology was adopted to define the different kinds of microtriches [41]. In our study, the microtriches observed among* A. centripunctata, M. expansa*, and* S. globipunctata* are acicular and capilliform filitriches. On the other hand, those among* T. ovilla* are capilliform filitriches. For a full species description of anoplocephalids, molecular analyses remain to be assessed. Previous isoenzyme electrophoretic studies have shown that* A. centripunctata, M. expansa*, and* T. ovilla* are species complex [14, 30]. But recently for* Moniezia expansa*, a genetic analysis showed an intraspecific variation other than speciation, and further molecular investigation is necessary, in order to identify species in all of these genera [42].

## 5. Conclusion

In small ruminants from Senegal, four cestode species* A. centripunctata, M. expansa, S. globipunctata*, and* T. ovilla* were found among sheep. Two,* M. expansa* and* T. ovilla*, were observed among goats. The prevalence of anoplocephalids was significantly higher among sheep than among goats. There were no statistically significant differences between dry and rainy season prevalence of Anoplocephalidae among sheep. Multiple infections of anoplocephalids were recorded and the double infection* A. centripunctata* and* S. globipunctata* was most prevalent with 24.2%.

## Figures and Tables

**Table 1:** Mean intensity of anoplocephalids among sheep during dry and rainy seasons.

	*A. centripunctata*	*M. expansa*	*S. globipunctata*	*T. ovilla*
Total	3.4 (4.0–1.8)	4.2 (9.0–1.5)	5.1 (12.5–1.0)	1.5 (2.0–1.0)
Dry season	3.7 (4.0–1.8)	4.9 (9.0–1.5)	5.4 (12.5–1.0)	2 (2.0–1.0)
Rainy season	3.2 (4.0–2.6)	3.6 (5.9–2.3)	4.9 (8.5–1.7)	1 (1.0–0.0)
